# The Role of Sphingolipid Metabolism in Bone Remodeling

**DOI:** 10.3389/fcell.2021.752540

**Published:** 2021-11-29

**Authors:** Tang Qi, Liao Li, Tian Weidong

**Affiliations:** State Key Laboratory of Oral Diseases, National Clinical Research Center for Oral Diseases, Engineering Research Center of Oral Translational Medicine, Ministry of Education, National Engineering Laboratory for Oral Regenerative Medicine, West China Hospital of Stomatology, West China School of Public Health, West China Fourth Hospital, Sichuan University, Chengdu, China

**Keywords:** sphingolipid, bone remodeling, osteoblast, osteoclast, S1P

## Abstract

Emerging studies of bioactive lipids have made many exciting discoveries in recent years. Sphingolipids and their metabolites perform a wide variety of cellular functions beyond energy metabolism. Emerging evidence based on genetically manipulated mouse models and molecular biology allows us to obtain new insights into the role sphingolipid played on skeletal remodeling. This review summarizes studies or understandings of the crosstalk between sphingomyelin, ceramide, and sphingosine-1-phosphate (S1P) of sphingolipids family and the cells, especially osteoblasts and osteoclasts of the bone through which bone is remodeled during life constantly. This review also shows agonists and antagonists of S1P as possible therapeutic options and opportunities on bone diseases.

## Background

Bone is an important tissue to provide biomechanical and structural supports to the body (Lee and Karsenty, 2008). Besides, it is a dynamic organ that undergoes bone formation and resorption. Bone is formed through two forms: endochondral and intramembranous ossification (Soltanoff et al., 2009), formation of which begins when mesenchymal cells adhere, and the osteogenesis can be made through the transformation of the pre-existing mesenchymal cells into bone tissue or sometimes by the replacement of the cartilage by bone (Zaidi, 2007). Then bone remodeling takes place after bone formation and development and continues during the whole lifetime (Kronenberg, 2003; Roberts et al., 2015; Serra-Vinardell et al., 2020). Development and lifelong remodeling of the bone involve some major bone-related cells, such as osteoblasts (Matsuo and Irie, 2008), osteoclast precursors, osteoclasts, osteocytes, bone lining cells, bone marrow stem cells (Lee et al., 2017), adipocytes, fibroblasts, immune cells (Arron and Choi, 2000; Pacifici, 2010, 2013; Rauner et al., 2013; Purdue et al., 2014; Pietschmann et al., 2016), and non-osteogenic cell populations by the blood supply. Their proliferation, differentiation, death, and dynamic balance determine the shape and function of bone. Besides, some other cellular systems, including cartilage, also play important roles. Among these cells, osteoblasts and osteoclasts play a critical role in bone remodeling. The maintenance of bone size, shape, integrity, and function of bone depends on the exquisite balance between osteoblasts and osteoclasts (Huang et al., 2007; Soltanoff et al., 2009; Matsuoka et al., 2014). Mainly osteoclasts are responsible for bone resorption, and osteoblasts are responsible for new bone formation. The imbalance can result in abnormal bone architecture or function; therefore, bone metabolism diseases will occur, such as osteoporosis and osteopetrosis (Zaidi, 2007). But osteoblasts and osteoclasts are closely connected through cytokine, cell-bone matrix contact, or direct cell-cell contact. Besides, the communication between osteoblasts and osteoclasts happens at various stages of bone remodeling (Matsuo and Irie, 2008). Lipid is an important nutrient of the body by offering energy, essential fatty acids (FAs), and other derivatives and influences many cell types, cell functions, and signaling pathways. Lipid could also be divided into eight categories, including fatty acyls, glycerolipid, glycerophospholipid, sphingolipid, sterol lipid, prenol lipid, saccharolipid, and polyketide. Each of them contains distinct classes and subclasses of molecules (Fahy et al., 2005; Liebisch et al., 2020). Lipid metabolism that is a complex process associated with biosynthesis and degradation controls the level of lipid. Lipid metabolism also involves the hydrolysis of lipid (Mu and Porsgaard, 2005) and then its hydrolysis product is absorbed, packaged, and transported to the rest of the cells or tissues.

**FIGURE 1:**
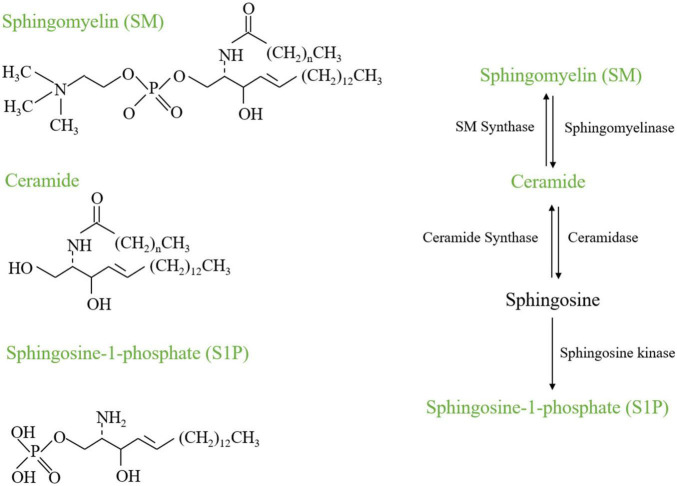
Chemical structures and metabolism of SM, Ceramide, and S1P.

Lipids play an important role in bone remodeling and bone disease. The study about the relationship between lipid metabolism and biomineralization was early in 1963 (Irving, 1963). The presence of lipid within a porous compartment of cortical bone restricts radial permeability, possibly influencing the metabolic functions of osteoblasts and osteocytes (Wen et al., 2010). Recently, a study found the obstruction of vascular invasion during bone healing tends to chondrogenic rather than osteogenic differentiation of skeletal progenitor cells, due to a decreased availability of extracellular lipids. Thus, lipid availability has been found to determine the fate of skeletal progenitor cells (van Gastel et al., 2020). It highlights that lipid plays a crucial role in a signal pathway, cell types, and functions in bone biology, and suggests its significance in bone pathology. Indeed, emerging data suggest the sphingolipid metabolism plays a critical role in skeletogenesis. Recent studies indicate the multifaceted influences of the sphingolipid on osteoblasts, osteoclasts, and the pivotal interaction underlying bone homeostasis-osteoblast and osteoclast crosstalk and highlight the multifaced roles of sphingolipid metabolism on bone remodeling. Hence, this review will focus on sphingolipid metabolism that regulates bone development and remodeling, involving the representative Sphingomyelin, Ceramide, and sphingosine 1-phosphate (Figure 1), teasing out their roles in the crosstalk of osteoblasts and osteoclasts.

## Sphingolipids Metabolism

Sphingolipids carry a long-chain sphingoid base with the 2-amino group amide-linked to a fatty acid, which forms ceramide, the core unit. Then different types of sphingolipids are formed by polar head groups added. The family of sphingolipids is defined by characters of the fatty acid, including carbon length, degree of unsaturation, and hydroxylation, along with other modifications of the LCBs and the polar head group (Hannun and Obeid, 2008; Teixeira and Costa, 2016). The sphingolipid metabolism shares a similar spatial organization which is highly conserved. It is governed by an integrated network of common synthetic and catabolic pathways that are modulated in response to different stimuli despite the diversity of sphingolipids (Hannun and Obeid, 2008; Teixeira and Costa, 2016).

**FIGURE 2:**
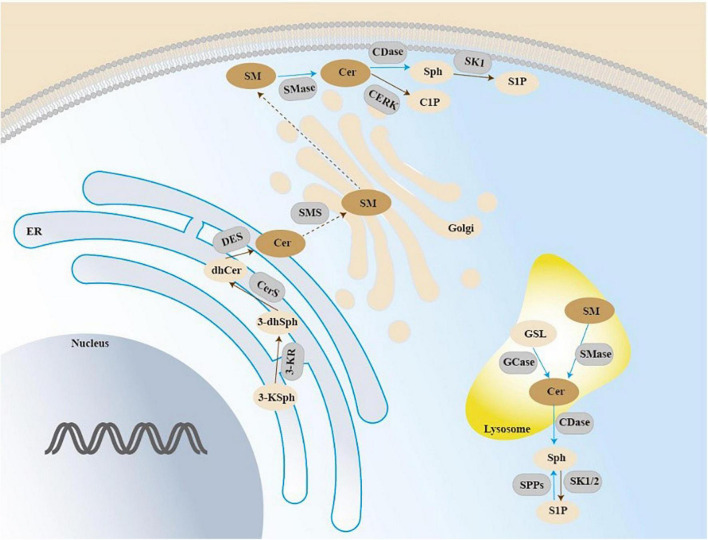
Sphingolipid pathways.

Sphingolipid biosynthesis *de novo* taking place in the endoplasmic reticulum (ER) involves a condensation reaction of serine and palmitoyl-CoA to produce 3-ketodihydrosphingosine catalyzed by serine palmitoyltransferase (SPT) (Merrill, 2002; Teixeira and Costa, 2016). 3-ketodihydrosphingosine generation is converted to the dihydroxyceramide (DHS) by ketodihydrosphingosine reductase (Kihara and Igarashi, 2004). DHS is acylated to dihydroceramides catalyzed by ceramide synthase (CERS1-6). Then dihydroceramide is catalyzed by dihydroceramide desaturases DES1 and DES2 to generate ceramide (Ternes et al., 2002). Ceramide serves as a substrate for enzymes that produce sphingolipids, sphingomyelin included. The ceramide is transported to the Golgi compartment by transfer protein CERT or vesicular, and then converted into sphingomyelin by sphingomyelin synthases (SMS). There are two isoforms of SMS, which is SMS1 and SMS2. SMS1 is only located in the Golgi apparatus while SMS2 is located in both the Golgi apparatus and the plasma membrane. These enzymes have been classified into three categories—acidic, alkaline, and neutral sphingomyelinases (Stoffel, 1999). The hydrolysis of ceramide generates sphingosine, which can generate sphingosine-1-phosphate (S1P) (Figure 2). The current review will discuss the role of some important sphingolipids in bone remodeling.

*De novo* sphingolipid biosynthesis begins at the endoplasmic reticulum (ER) with the condensation reaction of serine and palmitoyl-CoA forming 3-ketosphingosine. 3-ketodihydrosphingosine generation is converted to the dihydroxyceramide, and then acylated to dihydroceramides by ceramide synthase (CerS 1-6). Dihydroxyceramide is dehydrated between carbons 4 and 5 by dihydroceramide desaturase (DES) to form ceramide, and then be translocated to the Golgi. The action of SMS on ceramide results in the production of sphingomyelin (SM). Acid sphingomyelinase (SMase) is an enzyme converting sphingomyelin into ceramide. GSL can be transported intracellularly in the lysosome to generate ceramide. Ceramide can be generated through degradation of SM in the lysosome or at the plasma membrane by SMase. Then hydrolysis of ceramide by ceramidase generates sphingosine, which can be phosphorylated and generate sphingosine-1-phosphate (S1P).

## Sphingomyelin and Bone Remodeling

Sphingomyelin exists in diverse species, from protozoa to mammals. It is the major component of the double membrane-bound sphingolipids, which is generated from ceramide and phosphatidylcholine by sphingomyelin synthase and is implicated in cell survival, proliferation, migration, and inflammation (Tafesse et al., 2006; Taniguchi and Okazaki, 2014). However, its bioactive function mainly relies on its hydrolysis and downstream lipids, including ceramide and S1P. SM is essential for bone formation and normal mineralization. The abnormity of SM could cause defective bone mineralization, including osteoporosis, severe short stature, neonatal fractures, osteogenesis imperfecta, spondylometaphyseal dysplasia, severe bone and tooth mineralization defects, and gross skeletal abnormalities.

**(1) Sphingomyelin and bone formation**

Sphingomyelin is essential for bone formation. The local SM catabolism is found to be essential for the mineralization process in healthy bones. Catalyzing SM hydrolysis forms phosphocholine and ceramide, which are highly expressed in bone and are required for normal mineralization (During et al., 2015). Mutations in the sphingomyelin synthase 2 gene (SGMS2) could cause defective bone mineralization (Pekkinen et al., 2019). Patients with skeletal phenotypes and osteoporosis were identified with mutations in the SGMS2 gene encoding for the SMS2, with some sharing the same nonsense variant to yield a catalytically inactive enzyme and presenting with childhood-onset osteoporosis. In addition, others had a missense variant to enhance the rate of sphingomyelin production by blocking the export of a functional enzyme from the endoplasmic reticulum, with severe short stature, neonatal fractures, and spondylometaphyseal dysplasia. A recent case reported a family with moderately severe bone fragility and multiple sclerotic skull lesions similar to the osteogenesis imperfecta mentioned above; however, no pathogenic variant was found in SGMS2 (Makitie et al., 2021). Knocking down the SMS2 suppressed TRAP-positive multinucleated cells’ formation through co-culture of bone marrow cells and osteoblasts, which indicated that knockdown of SMS2 inhibits osteoclastogenesis through decreasing RANKL expression in primary osteoblasts of mice (Yoshikawa et al., 2019). In addition, the recent study found that based on metabolomic analysis, giant cell tumor of bone (GCTB), SM was checked as the most dysregulated phospholipid in GCTB, with high expression of SMS1 and SMS2, and low expression of nSMase2 (Quiroz-Acosta et al., 2021).

**(2) Sphingomyelin and bone resorption**

The excessive accumulation or catabolism of SM seemed to be linked to bone resorption. SMPD3 encodes neutral sphingomyelinase 2, the expression of which is restricted to the cartilage, bone, and brain (Khavandgar et al., 2011). Currently, there are two established SMPD3-deficient mouse models fro/fro model and SMPD3-/- model (Aubin et al., 2005; Stoffel et al., 2005). Both the two SMPD3-deficient mouse models show severe bone and tooth mineralization defects, and gross skeletal abnormalities. The fro mutation completely abolishes the enzymatic activity without affecting the location of SMPD3, thus reduction of nSMase activity in skeletal tissues marked by abnormal bone mineralization defects with high expression of SMPD3 is found in fro/fro mice (Khavandgar et al., 2011). Fro/fro mice also showed delayed mantle dentin mineralization and a consequent delay in enamel formation, but these tooth abnormalities progressively improved with time (Khavandgar et al., 2013). So far, no abnormalities of bone in mice lacking SMPD1 or SMPD2 activity have been reported. All of these revealed the excessive accumulation of SM properly could be tightly associated with bone defects. On the other side, although there is an increase of SM in bone marrow while there is a significant reduction of SM in the mineralized tissue part of OVX rat femurs (During et al., 2020), which suggested that excessive catabolism of SM could be associated with bone resorption. But more investigations will be needed regarding the potential role of SM in bone and the underlying mechanism.

## Ceramide and Bone Remodeling

Ceramide can arise from the endoplasmic reticulum by *de novo* synthesis (Hirschberg et al., 1993) and it can also be generated from the hydrolysis of SM by sphingomyelinases either at the plasma membrane or in endosomes or lysosomes. Ceramide is a bioactive lipid that serves as a second messenger in the regulation of cell death pathways and metabolism in response to stress, apoptotic triggers, and chemotherapy (Ryland et al., 2011; Kurek et al., 2013), involving extrinsic mechanism by mimicking the cytotoxicity of TNF (Hannun, 1994), and an intrinsic mechanism by modifying enzymes to regulate the level of ceramide (Morales et al., 2007), further leading to signal cascade and cell death by the downstream of Bcl2. However, S1P opposes the proapoptotic function of ceramide (Rutherford et al., 2013) and the ratio of S1P and ceramide is described as a rheostat of sphingolipid which is involved in the pathogenesis of certain cancers and this rheostat is one of the targets of anticancer drugs (Dyatlovitskaya et al., 2001). Ceramide plays an important role in bone metabolism. The abnormity of ceramide could cause osteoblast metabolic disorder and dysfunction, and thus influence the bone formation, and some special ceramides (C16:0, C18:0, C18:1, and C24:1) are correlated with bone resorption markers.

**(1) Ceramide and bone formation**

The alteration in the intracellular levels of ceramide could play a vital role in bone formation. C2-ceramide is reported to promote osteoblast viability, while high concentration (≥2 × 10^–6^M) reduces osteoblast viability. Increasing intracellular levels of ceramide also increase osteoblast apoptosis, determined by nuclear appearance and DNA fragmentation (Hill and Tumber, 2010). Endogenous cellular ceramide concentrations increase after TNF-α treatment, while the apoptosis of osteoblasts is triggered by TNF-α-generated ceramide by activating NF-κB signaling pathway. In addition, reducing the production of ceramide by dexamethasone inhibits TNF-α-induced activation of NF-κB and apoptosis in osteoblasts (Chae et al., 2000). Some external or internal stimuli impair the viability or physiological function of osteoblast through ceramide accumulation. Sodium nitroprusside enhances the release of intracellular ceramides C22 and C24 to decrease osteoblast viability (Olivier et al., 2005). Elevated palmitic acid intake significantly increases C16 ceramide accumulation and thus reduces osteoblast function *in vitro* and bone formation markers *in vivo* (Alsahli et al., 2016). In obese mice with palmitic acid or oleic acid-enriched high fat diet, ceramide accumulation in osteoblasts and suppresses bone formation (Alsahli et al., 2016). The influence of ceramide on osteoblast is in a dose- and time-dependent manner, and increasing levels of intracellular ceramide with either an inhibitor of ceramide metabolism or sphingomyelinase increased osteoblast apoptosis (Hill and Tumber, 2010).

**(2) Ceramide and bone resorption**

The role of ceramide in apoptosis is studied extensively, and recently ceramide is reported to be involved in bone cell survival, cell death, and bone resorption. However, at present, few experimental data directly link it with the mineralization of skeletal tissue. DES1 is one of the enzymes that form ceramide through the *de novo* pathway. The DES1 null mice show a normal skeletal structure, although they have multiple physiologic anomalies such as weight loss and growth impairment (Holland et al., 2007). The C24:1 ceramide in serum extracellular vesicles increases with age and could induce senescence in human bone marrow stromal cells (BMSCs) (Khayrullin et al., 2019). In patients 65 years or older with hip surgery, age was correlated with circulating levels of C16:0, C18:0, and C24:1 ceramide positively. Higher levels of C16:0, C18:0, C18:1, and C24:1 ceramide were positively related to bone resorption markers in both blood and bone marrow samples. C18:0 and C24:1 ceramide directly increased osteoclastogenesis *in vitro* (Kim et al., 2019). In support, the Postmenopausal Osteoporosis Mouse model found a significant reduction of three to five SM species and increased six metabolites, of which five were ceramide species (Zhao et al., 2018).

## Sphingosine-1-Phosphate and Bone Remodeling

Sphingosine-1-phosphate (S1P) is a natural lipid molecule that is formed by the phosphorylation of sphingosine and is derived from cell membrane sphingolipid (Spiegel and Milstien, 2002, 2003) as the product of sphingosine kinase(SK)1 and/or 2-mediated phosphorylation of sphingosine. In addition, S1P can either be converted back to sphingosine by specific S1P phosphatases or degraded by S1P lyase to form hexadecenal and phosphoethanolamine (Pebay et al., 2007). S1P is a common first or second messenger and serves as a mediator in regulating cell migration, death (Olivera and Spiegel, 1993), proliferation (Zhang et al., 1991; Cuvillier et al., 1996), and apoptosis (Cuvillier et al., 1996). Furthermore, it is involved in cell adhesion, cell motility, smooth muscle contraction, and platelet aggregation (Takuwa, 2002). It has been acquired in extensive study in cardiovascular, nervous, and immune systems, and its role in promoting angiogenesis is well-established (Alvarez et al., 2007). S1P acts either directly on intracellular targets or combines its known surface G-protein-coupled receptors S1P_1–5_ as a common second messenger. The binding of S1P to these receptors induces differential signaling pathway, and sometimes overlapping. S1P and its S1P_1–5_ receptors are expressed in variable systems, including vascular, immune, nervous, and reproductive systems (Hla, 2004). In recent years, S1P is implicated in osteogenesis-related processes, such as cell recruitment, cell differentiation, osteoblast survival, and coupling with osteoclasts.

S1P is important for osteoblast survival and the migration of osteoblasts and osteoclasts. The abnormity of S1P could cause osteopenia, reduced bone formation, and rheumatoid arthritis.

### Sphingosine-1-Phosphate and Bone Formation

#### Homing

Inhibiting S1P degradation or downregulate S1P_1_ receptors to dissipate the gradient between blood and bone marrow can reduce the number of circulating progenitor cells (Bendall and Basnett, 2013). The fractures in bone lead to increased S1P levels and hematopoietic stem cell migration (Golan et al., 2013). Various agents during mobilization are closely associated with the bone remodeling (Mendez-Ferrer et al., 2008), CXCR4 antagonist AMD3100 (Broxmeyer et al., 2005; Pusic and DiPersio, 2010) included. Using S1P lyase inhibitor to increase the bone marrow S1P concentration is shown to attenuate AMD3100-mediated progenitor cells mobilization in mice (Ratajczak et al., 2010; Golan et al., 2012; Juarez et al., 2012). Mice lacking SPK1 have impaired AMD3100-mediated progenitor cell mobilization, and besides, suppression of S1P_1_ receptor inhibits AMD3100-mediated progenitor cell mobilization (Golan et al., 2012; Juarez et al., 2012).

S1P stimulates mesenchymal (skeletal) cell chemotaxis by activating JAK/STATs and FAK/PI3K/AKT signaling pathways through S1P_1_ and S1P_2_ coordinately (Quint et al., 2013). S1P could be produced by osteoclast precursors during differentiation, and it enhances osteoblast survival in serum-deprived conditions (Ryu et al., 2006). In addition, it is a chemorepellent for pre-osteoblasts (Roelofsen et al., 2008), and increased osteoblast chemotaxis at the range of 0.01–1 μM.

#### Differentiation

As S1P could affect the migration of osteoblasts, additional efforts are made to know the role of S1P on the proliferation and differentiation of osteoblasts. S1P can act as an osteoanabolic molecule (Keller et al., 2014). The data from 4091 participants of the SHIP-Trend population-based study reveals a positive between serum levels of S1P, bone formation markers, and serum calcium, but not resorption markers. S1P participates in the proliferative process in human osteoblasts *via* MAP kinase activation (Carpio et al., 1999), and S1P-driven human osteoblast proliferation is predominantly linked to PKCα isoform (Lampasso et al., 2002).

For animals, in mice increasing S1P levels by conditionally deleting or inhibiting S1P lyase could increase bone formation, bone mass, and bone strength, and interestingly decreased white adipose tissue (Weske et al., 2018). It has been identified S1P receptors were in the key cells involved in bone remodeling, as S1P_1–3_ receptors are expressed in osteoblasts but S1P_4–5_ failed to be detected in primary osteoblasts (Grey et al., 2004), or much lower S1P_4_ receptor mRNA level and no detectable S1P_5_ receptor mRNA in osteoblasts (Keller et al., 2014). Besides, S1P_1_ and S1P_3_ receptors are increasing at the early stage of osteoblastogenesis. The Sgpl1^–/–^ mice, which lack the S1P lyase (Vogel et al., 2009; Liu et al., 2012), display high extracellular S1P levels and it causes various organ abnormalities, one of which the trabecular bone mass is remarkably increased at the age of 6 weeks. A study found that the S1P_1_-deficient mice died in *utero* (Allende et al., 2003), but S1P_3_-deficient mice do not display obvious abnormalities (Ishii et al., 2001). The S1P_3_-deficient mice at 3 months of age have no difference compared with wild mice, while the 8-month-old S1P_3_-deficient mice displayed osteopenia, reduced bone formation, and unaffected bone resorption parameters (Keller et al., 2014). But the bone mass or bone remodeling parameters are not an alteration in mice lacking S1P_1_ receptor specifically in osteoblasts both at 3 and 8 months. S1P_1_ and S1P_3_ are the candidate receptors controlling bone formation in response to S1P.

In primary rat osteoblasts, S1P is a potent osteoblast mitogen and the proliferative action of S1P is involved G_i_ protein, intracellular calcium, and p42/p44 MAP kinases (Grey et al., 2004). In C2C12 myoblasts, S1P receptor-mediated signaling plays a vital role in osteoblast differentiation by MEK1/2-ERK1/2 signaling pathway enhanced BMP-2-Smad signaling (Sato et al., 2012). In SaOS-2 and MC3T3-E1, two osteoblast-like cell lines, S1P activates the PI3K/Akt signaling pathway to the promotion of nuclear translocation of β-catenin in osteoblast-like cells, and upregulates osteoprotegerin and osteoblast differentiation markers (Matsuzaki et al., 2013). SphK1 is expressed in human and mouse osteoblastic cells, which secrete a large amount of S1P, and the process is accompanied by decreased levels of S1P_1_ and S1P_2_, but increased levels of S1P_3_. The autocrine S1P/S1P_3_ signaling is a core signaling pathway during differentiation to mature osteoblasts by regulating runx2, which plays a key role in transcription factor associated with osteoblast differentiation and osteoblastic maturation (Brizuela et al., 2014). S1P significantly increases matrix mineralization in wild-type mice and a rapid phosphorylation Erk1/2, while both are not detected in S1P_3_ receptor-deficient mice during osteogenesis. In addition, during the process, S1P negatively regulated S1P_3_ receptor in wild-type cultures.

### Sphingosine-1-Phosphate and Bone Resorption

#### Homing

Some causes of bone disease depend on the recruitment of osteoclasts into resorption sites or due to the migration of inflammatory cells by chemokine gradients. S1P promotes the entry of osteoclast precursors into the bone from blood and promotes osteoclast differentiation (Ishii et al., 2009, 2010). OP-positive chemotaxis is prominent in gradients with low maximal concentrations of S1P and with high maximal S1P concentrations, Cells with properties of osteoclast precursors express S1P_1_ receptors, and using S1P_1_ agonist SEW2871 can stimulate motility of osteoclast precursor-containing monocytoid populations. In addition, OC/monocyte (CD11b) lineage-specific conditional S1P_1_ receptor knockout mice increased osteoclasts attaching to the bone surface to make bone osteoporosis changes (Ishii et al., 2009), and Gi and Rac are involved in S1P_1_ receptor-mediated chemoattraction. While osteoclast precursors also express S1P_2_, which mediates negative chemotaxis of osteoclast precursors. The S1P_2_-mediated chemorepulsion overrides S1P_1_ upgradient motion. S1P_2_ inhibited the chemotaxis of BMMs by treatment with S1P_2_ siRNA. The combined results indicate that cell migration controlled by S1P relies on the gradient between tissues, the doses of S1P, or the balance between S1P and other chemokines.

#### Differentiation

Bone loss in many diseases, including osteoporosis, rheumatoid arthritis etc. (Redlich and Smolen, 2012), is featured with proinflammatory cytokines, and RANKL is the most important molecule among them to regulate OC differentiation. In addition, inflammatory conditions are always associated with high levels of S1P (Lee et al., 2012). It shows that postmenopausal women had higher S1P plasma levels, and positively correlated with low bone mineral density, compared to premenopausal women and men (Lee et al., 2012). The SK1 deficiency in mice alleviated periodontal alveolar bone loss, and S1P dose-dependently increased chemotaxis of murine bone marrow-derived monocytes. It is also shown a significantly higher level of S1P in synovial fluid of patients with rheumatoid arthritis (Lai et al., 2012). In addition, SK1 deficiency in mice decreased inflammation and joint erosions in murine arthritis (Baker et al., 2010).

S1P is a coupling factor between osteoclasts and osteoblasts. It impacts OC precursor differentiation by regulating RANKL or its downstream signaling pathway. Increased S1P production and secretion and upregulated SPHK1 expression are observed in a bone marrow-derived macrophage model system by RANKL stimulation. The osteoclastogenesis is greatly increased by adding S1P to BMM/osteoblast co-culture system, indicating that S1P affects the osteoclastogenesis (Ryu et al., 2006). One of the important mechanisms for bone resorption is that osteoclast-secreted S1P increases RANKL. However, deletion of cathepsin k in osteoclasts, which is secreted by osteoclasts to degrade collagen and other matrix proteins, increased the SPHK1 expression, and conditioned media from cathepsin k-deficient osteoclasts, in which the levels of S1P elevated, increased alkaline phosphatase and mineralized nodules in osteoblast culture (Lotinun et al., 2013). Sphingosylphosphorylcholine (SPC), a biological lipid that can be converted to S1P by autotaxin and share receptors with S1P, is reported to inhibit RANKL-induced osteoclast differentiation. But SPC-induced inhibitory effects are not altered by several antagonists of S1P receptors (Lee et al., 2021), suggesting the independence of S1P and SPC on surface receptors, and thus denying the speculation of receptor competition.

S1P_1–2_ receptors are detected in osteoclast precursor cells and mRNA for all S1P receptors except S1P_5_ in bone marrow-derived macrophages and differentiating osteoclasts (Ryu et al., 2006; Keller et al., 2014). S1P_2_ seems closely linked to osteoclasts. S1P_2_ played an important role in regulating proinflammatory cytokine release induced by the oral bacterial pathogen Aa. shRNA of S1P_2_ reduced IL-1β, IL-6, and TNF-α levels in BMMs induced by Aa. In addition, knockdown of S1P_2_ suppressed p-PI3K, p-ERK, p-JNK, p-p38 MAPK, and p-NF-κBp65 levels induced by Aa. Furthermore, knockdown of S1P_2_ significantly suppressed factors associated with osteoclast formation/activity, including the nuclear factor of activated T-cells cytoplasmic calcineurin-dependent 1(Nfatc 1), acid phosphatase 5 (Acp5), cathepsin K(Ctsk), osteoclast-associated receptor (Oscar) (Yu, 2016). S1P_2_-deficient mice exhibit moderate osteopetrosis because of a decrease in osteoclastic bone resorption. Using S1P_2_ antagonist JTE013 can change the migration of osteoclast precursors and relieved osteoporosis in a mice model by limiting op localization and reduced osteoclasts (Ishii et al., 2010). However, another study showed S1P_2_-deficient mice were osteopenic and obese. S1P signaling through S1P_2_ potently stimulated osteoblastogenesis by inversely regulating osterix and PPAR-γ at the expense of adipogenesis, and simultaneously the osteoclastogenesis is inhibited through p38-GSK3β-β-catenin and WNT5a-LRP5 pathway (Weske et al., 2018).

## Treatment of Bone Disease by Targeting Sphingomyelin and Downstream Pathway

The appropriate level of sphingomyelin without excessive anabolism or catabolism plays an essential role in bone remodeling and prevents bone diseases. Adjusting the level of sphingomyelin in bone is the targeting treatment by activating the SMS or SMase. Sphingomyelin performed its function by its hydrolysis. While it lacks adequate studies of Ceramide, more research about S1P on bone disease is needed, thus introducing some possible treatments about S1P on bone diseases.

### Agonists

FTY720 is a mimetic of natural sphingosine and therefore can be recognized by part of the cellular sphingosine enzymatic machinery (Zemann et al., 2006; Mechtcheriakova et al., 2007). It is suggested that FT720 is a prodrug and FTY720-P is its phosphorylation by SphKs, most efficiently by SphK2, can act as a mimetic of S1P as an S1P receptor agonist (Billich et al., 2003) and specifically binds to four out of five S1P receptors, except S1P_2_ (Brinkmann et al., 2002; Spiegel and Milstien, 2011). It is described that daily injection of the nonselective S1P receptor agonist FTY720 protects against ovariectomy-induced bone loss (Ishii et al., 2009). In addition, the trabecular bone volume is increased in wild-type mice treatment with FTY720 daily, whereas S1P_3_-deficient mice do not respond (Keller et al., 2014). Although FTY720-P is an agonist of S1P_1/3/4/5_, its effects are inhibitory on S1P receptor function in the longer term. The mechanism of its antagonism function is suggested to be associated with receptor internalization and in part is based on the ability to target the S1P_1_ receptor to the proteasomal degradation pathway through poly-ubiquitination (Oo et al., 2007). Treatment with FTY720 relieved ovariectomy-induced osteoporosis by facilitating recirculation of osteoclast precursor-containing cell populations and reducing the number of mature osteoclasts attached to the bone in mice (Ishii et al., 2009). KPR-203 has a structural similarity with FTY720. KPR203 and FTY720 both have a similar high affinity for the S1P_1_ receptor. To date, the effect of KRP-203 on bone remodeling has not yet been reported. SEW2871 is an S1P_1_ receptor-selective agonist, and not active for the S1P_2–5_ receptors unlike FTY720 of a nonselective S1P receptor agonist. SEW2871 can induce the recruitment of macrophages (Lien et al., 2006; Takabe et al., 2008), and the hydrogels incorporating mixed SEW2871 and PRP promoted bone regeneration to a great extent, which suggests macrophage recruitment contributed to PRP-induced bone regeneration (Kim et al., 2014). Cells with the properties of osteoclast precursors express S1P_1_ receptors and exhibit positive chemotaxis along an S1P gradient *in vitro*. In addition, intravital two-photon imaging of bone tissues showed that SEW2871 stimulated motility of osteoclast precursor-containing monocytoid populations *in vivo*. Osteoclast/monocyte lineage-specific conditional S1P_1_ knockout mice showed osteoporotic changes due to increased osteoclast attachment to the bone surface (Ishii et al., 2009).

### Antagonist

VPC23019 is an unselective S1P_1_ and S1P_3_ antagonist. Targeted ablation of cathepsin K which is secreted by osteoclasts to degrade collagen and other matrix proteins during bone resorption, in hematopoietic cells, and specifically in osteoclasts and cells of monocyte-osteoclast lineage causes increased bone volume and bone formation rate. In contrast, the targeted deletion of cathepsin K in osteoblasts did not get those results. The deletion of cathepsin K in osteoclasts increases SK1 expression. Conditioned media from cathepsin K-deficient osteoclasts with elevated levels of S1P increased alkaline phosphatase and mineralized nodules in osteoblast culture with an increased RANKL/OPG ratio. However, VPC 23019 inhibited these process (Lotinun et al., 2013). JTE013, a specific and most used competitive S1P_2_ receptor antagonist, was synthesized at the Central Pharmaceutical Institute in Japan in 2001 (Takabe et al., 2008). It was shown to antagonize the binding of radiolabeled S1P in Chinese hamster ovary cells overexpressing S1P_2_ receptor (Pyne and Pyne, 2011). JTE013 suppresses PI3K, MAPKs, and NF-κB and inhibits the release of IL-1β,IL-6,TNF-α, and S1P in murine bone marrow cells. In addition, JTE013 suppressed osteoclastogenesis and bone resorption through changing monocyte migration behavior induced by RANKL in murine bone marrow cultures (Hsu et al., 2019).

## Conclusion

Although the published studies have displayed the critical role of sphingomyelin metabolism in osteoblasts, osteoclasts, and bone remodeling, the studies on the mechanism are still few. The sphingomyelin is essential in bone formation, but the excessive accumulation or catabolism of SM properly could be tightly associated with bone resorption. At present, limited direct evidence is available on the roles of sphingomyelin in osteoblasts and osteoclasts, and further, the enzymes of sphingomyelin are little studied in the bone tissue. As for Ceramide, despite the pro-apoptosis function in osteoblasts, the links of the differentiation of osteoblasts to ceramide are still unknown. In addition, recently, osteoclast can be mediated by ceramide. The comparatively sufficient studies of S1P on bone remodeling allow us to further study the treatment of associated bone diseases, although the role of S1P is complicated and depending on the different receptors of S1P_1–5_.

## Author Contributions

TW and LL conceived of the presented idea. TQ finished the article. All authors contributed to the article and approved the submitted version.

## Conflict of Interest

The authors declare that the research was conducted in the absence of any commercial or financial relationships that could be construed as a potential conflict of interest.

## Publisher’s Note

All claims expressed in this article are solely those of the authors and do not necessarily represent those of their affiliated organizations, or those of the publisher, the editors and the reviewers. Any product that may be evaluated in this article, or claim that may be made by its manufacturer, is not guaranteed or endorsed by the publisher.

## References

[B1] Allende M. L. Yamashita T. Proia R. L. G. (2003). Protein-coupled receptor S1p1 acts within endothelial cells to regulate vascular maturation. *Blood* 102 3665–3667. 10.1182/blood-2003-02-0460 12869509

[B2] Alsahli A. Kiefhaber K. Gold T. Muluke M. Jiang H. Cremers S. (2016). Palmitic acid reduces circulating bone formation markers in obese animals and impairs osteoblast activity *Via* C16-ceramide accumulation. *Calcif. Tissue Int.* 98 511–519. 10.1007/s00223-015-0097-z 26758875

[B3] Alvarez S. E. Milstien S. Spiegel S. (2007). Autocrine and paracrine roles of sphingosine-1-phosphate. *Trends Endocrinol. Metab.* 18 300–307. 10.1016/j.tem.2007.07.005 17904858

[B4] Arron J. R. Choi Y. (2000). Bone versus immune system. *Nature* 408 535–536. 10.1038/35046196 11117729

[B5] Aubin I. Adams C. P. Opsahl S. Septier D. Bishop C. E. Auge N. (2005). Deletion in the gene encoding sphingomyelin phosphodiesterase 3 (Smpd3) results in osteogenesis and dentinogenesis imperfecta in the mouse. *Nat. Genet.* 37 803–805. 10.1038/ng1603 16025116

[B6] Baker D. A. Barth J. Chang R. Obeid L. M. Gilkeson G. S. (2010). Genetic sphingosine kinase 1 deficiency significantly decreases synovial inflammation and joint erosions in murine tnf-alpha-induced arthritis. *J. Immunol.* 185 2570–2579. 10.4049/jimmunol.1000644 20644167 PMC2942019

[B7] Bendall L. J. Basnett J. (2013). Role of sphingosine 1-phosphate in trafficking and mobilization of hematopoietic stem cells. *Curr. Opin. Hematol.* 20 281–288. 10.1097/moh.0b013e3283606090 23507960

[B8] Billich A. Bornancin F. Devay P. Mechtcheriakova D. Urtz N. Baumruker T. (2003). Phosphorylation of the immunomodulatory drug Fty720 by sphingosine kinases. *J. Biol. Chem.* 278 47408–47415. 10.1074/jbc.m307687200 13129923

[B9] Brinkmann V. Davis M. D. Heise C. E. Albert R. Cottens S. Hof R. (2002). The immune modulator fty720 targets sphingosine 1-phosphate receptors. *J. Biol. Chem.* 277 21453–21457. 10.1074/jbc.c200176200 11967257

[B10] Brizuela L. Martin C. Jeannot P. Ader I. Gstalder C. Andrieu G. (2014). Osteoblast-derived sphingosine 1-phosphate to induce proliferation and confer resistance to therapeutics to bone metastasis-derived prostate cancer cells. *Mol. Oncol.* 8 1181–1195. 10.1016/j.molonc.2014.04.001 24768038 PMC5528572

[B11] Broxmeyer H. E. Orschell C. M. Clapp D. W. Hangoc G. Cooper S. Plett P. A. (2005). Rapid mobilization of murine and human hematopoietic stem and progenitor cells with amd3100, a cxcr4 antagonist. *J. Exp. Med.* 201 1307–1318. 10.1084/jem.20041385 15837815 PMC2213145

[B12] Carpio L. C. Stephan E. Kamer A. Dziak R. (1999). Sphingolipids stimulate cell growth *via* map kinase activation in osteoblastic cells. *Prostaglandins Leukot. Essent. Fatty Acids* 61 267–273. 10.1054/plef.1999.0100 10670688

[B13] Chae H. J. Chae S. W. Kang J. S. Bang B. G. Cho S. B. Park R. K. (2000). Dexamethasone suppresses tumor necrosis factor-alpha-induced apoptosis in osteoblasts: possible role for ceramide. *Endocrinology* 141 2904–2913. 10.1210/endo.141.8.7604 10919278

[B14] Cuvillier O. Pirianov G. Kleuser B. Vanek P. G. Coso O. A. Gutkind S. (1996). Suppression of ceramide-mediated programmed cell death by sphingosine-1-phosphate. *Nature* 381 800–803. 10.1038/381800a0 8657285

[B15] During A. Coutel X. Bertheaume N. Penel G. Olejnik C. (2020). Long term ovariectomy-induced osteoporosis is associated with high stearoyl-coa desaturase indexes in rat femur. *Calcif. Tissue Int.* 106 315–324. 10.1007/s00223-019-00637-7 31796982

[B16] During A. Penel G. Hardouin P. (2015). Understanding the local actions of lipids in bone physiology. *Prog. Lipid Res.* 59 126–146. 10.1016/j.plipres.2015.06.002 26118851

[B17] Dyatlovitskaya E. V. Kandyba A. G. Kozlov A. M. Somova O. G. (2001). Sphinganine in sphingomyelins of tumors and mouse regenerating liver. *Biochem. Biokhim.* 66 502–504. 10.1023/a:1010250600604 11405884

[B18] Fahy E. Subramaniam S. Brown H. A. Glass C. K. Merrill A. H. Jr. Murphy R. C. (2005). Comprehensive classification system for lipids. *J. Lipid Res.* 46 839–861. 15722563 10.1194/jlr.E400004-JLR200

[B19] Golan K. Kollet O. Lapidot T. (2013). Dynamic cross talk between s1p and cxcl12 regulates hematopoietic stem cells migration, development and bone remodeling. *Pharmaceuticals* 6 1145–1169. 10.3390/ph6091145 24276423 PMC3818832

[B20] Golan K. Vagima Y. Ludin A. Itkin T. Cohen-Gur S. Kalinkovich A. (2012). S1p promotes murine progenitor cell egress and mobilization *via* s1p1-mediated ros signaling and sdf-1 release. *Blood* 119 2478–2488. 10.1182/blood-2011-06-358614 22279055 PMC4017293

[B21] Grey A. Xu X. Hill B. Watson M. Callon K. Reid I. R. (2004). Osteoblastic cells express phospholipid receptors and phosphatases and proliferate in response to sphingosine-1-phosphate. *Calcif. Tissue Int.* 74 542–550. 10.1007/s00223-003-0155-9 15354862

[B22] Hannun Y. A. (1994). The sphingomyelin cycle and the second messenger function of ceramide. *J. Biol. Chem.* 269 3125–3128. 10.1016/s0021-9258(17)41834-5 8106344

[B23] Hannun Y. A. Obeid L. M. (2008). Principles of bioactive lipid signalling: lessons from sphingolipids. *Nat. Rev. Mol. Cell Biol.* 9 139–150. 10.1038/nrm2329 18216770

[B24] Hill P. A. Tumber A. (2010). Ceramide-induced cell death/survival in murine osteoblasts. *J. Endocrinol.* 206 225–233. 10.1677/JOE-10-0068 20466846

[B25] Hirschberg K. Rodger J. Futerman A. H. (1993). The long-chain sphingoid base of sphingolipids is acylated at the cytosolic surface of the endoplasmic reticulum in rat liver. *Biochem. J.* 290 (Pt 3) 751–757. 10.1042/bj2900751 8457204 PMC1132344

[B26] Hla T. (2004). Physiological and pathological actions of sphingosine 1-phosphate. *Semin. Cell Dev. Biol.* 15 513–520. 10.1016/j.semcdb.2004.05.002 15271296

[B27] Holland W. L. Brozinick J. T. Wang L. P. Hawkins E. D. Sargent K. M. Liu Y. (2007). Inhibition of ceramide synthesis ameliorates glucocorticoid-, saturated- fat-, and obesity-induced insulin resistance. *Cell Metab.* 5 167–179. 10.1016/j.cmet.2007.01.002 17339025

[B28] Hsu L. C. Reddy S. V. Yilmaz O. Yu H. (2019). Sphingosine-1-phosphate receptor 2 controls podosome components induced by rankl affecting osteoclastogenesis and bone resorption. *Cells* 8:17. 10.3390/cells8010017 30609675 PMC6357083

[B29] Huang W. Yang S. Shao J. Li Y. P. (2007). Signaling and transcriptional regulation in osteoblast commitment and differentiation. *Front. Biosci.* 12:3068–3092. 10.2741/2296 17485283 PMC3571113

[B30] Irving J. T. (1963). Calcification of the organic matrix of enamel. *Arch. Oral Biol.* 8 773–774. 10.1016/0003-9969(63)90010-4 14081617

[B31] Ishii I. Friedman B. Ye X. Kawamura S. McGiffert C. Contos J. J. (2001). Selective loss of sphingosine 1-phosphate signaling with no obvious phenotypic abnormality in mice lacking its g protein-coupled receptor, lp(b3)/edg-3. *J. Biol. Chem.* 276 33697–33704. 10.1074/jbc.M104441200 11443127

[B32] Ishii M. Egen J. G. Klauschen F. Meier-Schellersheim M. Saeki Y. Vacher J. (2009). Sphingosine-1-phosphate mobilizes osteoclast precursors and regulates bone homeostasis. *Nature* 458 524–528. 10.1038/nature07713 19204730 PMC2785034

[B33] Ishii M. Kikuta J. Shimazu Y. Meier-Schellersheim M. Germain R. N. (2010). Chemorepulsion by blood S1p regulates osteoclast precursor mobilization and bone remodeling *in vivo*. *J. Exp. Med.* 207 2793–2798. 10.1084/jem.20101474 21135136 PMC3005230

[B34] Juarez J. G. Harun N. Thien M. Welschinger R. Baraz R. Pena A. D. (2012). Sphingosine-1-phosphate facilitates trafficking of hematopoietic stem cells and their mobilization by cxcr4 antagonists in mice. *Blood* 119 707–716. 10.1182/blood-2011-04-348904 22049516

[B35] Keller J. Catala-Lehnen P. Huebner A. K. Jeschke A. Heckt T. Lueth A. (2014). Calcitonin controls bone formation by inhibiting the release of sphingosine 1-phosphate from osteoclasts. *Nat. Commun.* 5:5215. 10.1038/ncomms6215 25333900 PMC4205484

[B36] Khavandgar Z. Alebrahim S. Eimar H. Tamimi F. McKee M. D. Murshed M. (2013). Local regulation of tooth mineralization by sphingomyelin phosphodiesterase 3. *J. Dent. Res.* 92 358–364. 10.1177/0022034513478429 23428435

[B37] Khavandgar Z. Poirier C. Clarke C. J. Li J. Wang N. McKee M. D. (2011). Cell-autonomous requirement for neutral sphingomyelinase 2 in bone mineralization. *J. Cell Biol.* 194 277–289. 10.1083/jcb.201102051 21788370 PMC3144407

[B38] Khayrullin A. Krishnan P. Martinez-Nater L. Mendhe B. Fulzele S. Liu Y. (2019). Very long-chain c24:1 ceramide is increased in serum extracellular vesicles with aging and can induce senescence in bone-derived mesenchymal stem cells. *Cells* 8:37. 10.3390/cells8010037 30634626 PMC6356348

[B39] Kihara A. Igarashi Y. (2004). Fvt-1 is a mammalian 3-ketodihydrosphingosine reductase with an active site that faces the cytosolic side of the endoplasmic reticulum membrane. *J. Biol. Chem.* 279 49243–49250. 10.1074/jbc.M405915200 15328338

[B40] Kim B. J. Lee J. Y. Park S. J. Lee S. H. Kim S. J. Yoo H. J. (2019). Elevated ceramides 18:0 and 24:1 with aging are associated with hip fracture risk through increased bone resorption. *Aging* 11 9388–9404. 10.18632/aging.102389 31675352 PMC6874435

[B41] Kim Y. H. Furuya H. Tabata Y. (2014). Enhancement of bone regeneration by dual release of a macrophage recruitment agent and platelet-rich plasma from gelatin hydrogels. *Biomaterials* 35 214–224. 10.1016/j.biomaterials.2013.09.103 24125774

[B42] Kronenberg H. M. (2003). Developmental regulation of the growth plate. *Nature* 423 332–336. 10.1038/nature01657 12748651

[B43] Kurek K. Lukaszuk B. Piotrowska D. M. Wiesiolek P. Chabowska A. M. Zendzian-Piotrowska M. (2013). Metabolism, physiological role, and clinical implications of sphingolipids in gastrointestinal tract. *BioMed Res. Int.* 2013:908907. 24083248 10.1155/2013/908907 PMC3780527

[B44] Lai W. Q. Chia F. L. Leung B. P. (2012). Sphingosine kinase and sphingosine-1-phosphate receptors: novel therapeutic targets of rheumatoid arthritis? *Future Med. Chem.* 4 727–733. 10.4155/fmc.12.28 22530637

[B45] Lampasso J. D. Marzec N. Margarone J. III Dziak R. (2002). Role of protein kinase C alpha in primary human osteoblast proliferation. *J. Bone Mineral Res.* 17 1968–1976. 10.1359/jbmr.2002.17.11.1968 12412804

[B46] Lee H. Y. Cho K. M. Kim M. K. Lee M. Kim H. Choi C. Y. (2021). Sphingosylphosphorylcholine blocks ovariectomy-induced bone loss by suppressing Ca(2+) /calmodulin-mediated osteoclast differentiation. *J. Cell. Mol. Med.* 25 473–483. 10.1111/jcmm.16101 33230972 PMC7810965

[B47] Lee N. K. Karsenty G. (2008). Reciprocal regulation of bone and energy metabolism. *Trends Endocrinol. Metab.* 19 161–166. 10.1016/j.tem.2008.02.006 18407515

[B48] Lee S. H. Lee S. Y. Lee Y. S. Kim B. J. Lim K. H. Cho E. H. (2012). Higher circulating sphingosine 1-phosphate levels are associated with lower bone mineral density and higher bone resorption marker in humans. *J. Clin. Endocrinol. Metab.* 97 E1421–E1428. 22679064 10.1210/jc.2012-1044

[B49] Lee W. C. Guntur A. R. Long F. Rosen C. J. (2017). Energy metabolism of the osteoblast: implications for osteoporosis. *Endocr. Rev.* 38 255–266. 10.1210/er.2017-00064 28472361 PMC5460680

[B50] Liebisch G. Fahy E. Aoki J. Dennis E. A. Durand T. Ejsing C. S. (2020). Update on lipid maps classification, nomenclature, and shorthand notation for ms-derived lipid structures. *J. Lipid Res.* 61 1539–1555. 10.1194/jlr.S120001025 33037133 PMC7707175

[B51] Lien Y. H. Yong K. C. Cho C. Igarashi S. Lai L. W. (2006). S1p(1)-selective agonist, sew2871, ameliorates ischemic acute renal failure. *Kidney Int.* 69 1601–1608. 10.1038/sj.ki.5000360 16572108

[B52] Liu X. Zhang Q. H. Yi G. H. (2012). Regulation of metabolism and transport of sphingosine-1-phosphate in mammalian cells. *Mol. Cell. Biochem.* 363 21–33. 10.1007/s11010-011-1154-1 22113622

[B53] Lotinun S. Kiviranta R. Matsubara T. Alzate J. A. Neff L. Luth A. (2013). Osteoclast-specific cathepsin k deletion stimulates s1p-dependent bone formation. *J. Clin. Investig.* 123 666–681. 10.1172/JCI64840 23321671 PMC3561821

[B54] Makitie R. E. Pekkinen M. Morisada N. Kobayashi D. Yonezawa Y. Nishimura G. (2021). Novel ifitm5 variant associated with phenotype of osteoporosis with calvarial doughnut lesions: a case report. *Calcif. Tissue Int.* 109 626–632. 10.1007/s00223-021-00878-5 34156493 PMC8531111

[B55] Matsuo K. Irie N. (2008). Osteoclast-osteoblast communication. *Arch. Biochem. Biophys.* 473 201–209. 10.1016/j.abb.2008.03.027 18406338

[B56] Matsuoka K. Park K. A. Ito M. Ikeda K. Takeshita S. (2014). Osteoclast-derived complement component 3a stimulates osteoblast differentiation. *J. Bone Mineral Res.* 29 1522–1530. 10.1002/jbmr.2187 24470120

[B57] Matsuzaki E. Hiratsuka S. Hamachi T. Takahashi-Yanaga F. Hashimoto Y. Higashi K. (2013). Sphingosine-1-phosphate promotes the nuclear translocation of beta-catenin and thereby induces osteoprotegerin gene expression in osteoblast-like cell lines. *Bone* 55 315–324. 10.1016/j.bone.2013.04.008 23612487

[B58] Mechtcheriakova D. Wlachos A. Sobanov J. Bornancin F. Zlabinger G. Baumruker T. (2007). Fty720-phosphate is dephosphorylated by lipid phosphate phosphatase 3. *FEBS Lett.* 581 3063–3068. 10.1016/j.febslet.2007.05.069 17555747

[B59] Mendez-Ferrer S. Lucas D. Battista M. Frenette P. S. (2008). Haematopoietic stem cell release is regulated by circadian oscillations. *Nature* 452 442–447. 10.1038/nature06685 18256599

[B60] Merrill A. H. Jr. (2002). De novo sphingolipid biosynthesis: a necessary, but dangerous, pathway. *J. Biol. Chem.* 277 25843–25846. 10.1074/jbc.R200009200 12011104

[B61] Morales A. Lee H. Goni F. M. Kolesnick R. Fernandez-Checa J. C. (2007). Sphingolipids and cell death. *Apoptosis* 12 923–939. 17294080 10.1007/s10495-007-0721-0

[B62] Mu H. Porsgaard T. (2005). The metabolism of structured triacylglycerols. *Prog. Lipid Res.* 44 430–448. 10.1016/j.plipres.2005.09.002 16269186

[B63] Olivera A. Spiegel S. (1993). Sphingosine-1-phosphate as second messenger in cell proliferation induced by pdgf and fcs mitogens. *Nature* 365 557–560. 10.1038/365557a0 8413613

[B64] Olivier S. Fillet M. Malaise M. Piette J. Bours V. Merville M. P. (2005). Sodium nitroprusside-induced osteoblast apoptosis is mediated by long chain ceramide and is decreased by raloxifene. *Biochem. Pharmacol.* 69 891–901. 10.1016/j.bcp.2004.11.030 15748701

[B65] Oo M. L. Thangada S. Wu M. T. Liu C. H. Macdonald T. L. Lynch K. R. (2007). Immunosuppressive and anti-angiogenic sphingosine 1-phosphate receptor-1 agonists induce ubiquitinylation and proteasomal degradation of the receptor. *J. Biol. Chem.* 282 9082–9089. 10.1074/jbc.M610318200 17237497

[B66] Pacifici R. (2013). Osteoimmunology and its implications for transplantation. *Am. J. Transplant.* 13 2245–2254. 10.1111/ajt.12380 23915249

[B67] Pacifici R. T. (2010). Cells: critical bone regulators in health and disease. *Bone* 47 461–471. 10.1016/j.bone.2010.04.611 20452473 PMC2926258

[B68] Pebay A. Bonder C. S. Pitson S. M. (2007). Stem cell regulation by lysophospholipids. *Prostaglandins* & *Other Lipid Mediat.* 84 83–97. 10.1016/j.prostaglandins.2007.08.004 17991611

[B69] Pekkinen M. Terhal P. A. Botto L. D. Henning P. Makitie R. E. Roschger P. (2019). Osteoporosis and skeletal dysplasia caused by pathogenic variants in sgms2. *JCI Insight* 4:e126180. 10.1172/jci.insight.126180 30779713 PMC6483641

[B70] Pietschmann P. Mechtcheriakova D. Meshcheryakova A. Foger-Samwald U. Ellinger I. (2016). Immunology of osteoporosis: a mini-review. *Gerontology* 62 128–137. 10.1159/000431091 26088283 PMC4821368

[B71] Purdue P. E. Crotti T. N. Shen Z. Swantek J. Li J. Hill J. (2014). Comprehensive profiling analysis of actively resorbing osteoclasts identifies critical signaling pathways regulated by bone substrate. *Sci. Rep.* 4:7595. 10.1038/srep07595 25534583 PMC4274512

[B72] Pusic I. DiPersio J. F. (2010). Update on clinical experience with amd3100, an sdf-1/cxcl12-cxcr4 inhibitor, in mobilization of hematopoietic stem and progenitor cells. *Curr. Opin. Hematol.* 17 319–326. 10.1097/MOH.0b013e328338b7d5 20473162

[B73] Pyne N. J. Pyne S. (2011). Selectivity and specificity of sphingosine 1-phosphate receptor ligands: “off-targets” or complex pharmacology? *Front. Pharmacol.* 2:26. 10.3389/fphar.2011.00026 21687518 PMC3108476

[B74] Quint P. Ruan M. Pederson L. Kassem M. Westendorf J. J. Khosla S. (2013). Sphingosine 1-phosphate (s1p) receptors 1 and 2 coordinately induce mesenchymal cell migration through s1p activation of complementary kinase pathways. *J. Biol. Chem.* 288 5398–5406. 10.1074/jbc.M112.413583 23300082 PMC3581421

[B75] Quiroz-Acosta T. Flores-Martinez Y. M. Becerra-Martinez E. Perez-Hernandez E. Perez-Hernandez N. Banuelos-Hernandez A. E. (2021). Aberrant sphingomyelin (31)p-nmr signatures in giant cell tumour of bone. *Biochem. Cell Biol. Biochim. Biol. Cell.* 5, 1–8. 10.1139/bcb-2020-0599 34096319

[B76] Ratajczak M. Z. Lee H. Wysoczynski M. Wan W. Marlicz W. Laughlin M. J. (2010). Novel insight into stem cell mobilization-plasma sphingosine-1-phosphate is a major chemoattractant that directs the egress of hematopoietic stem progenitor cells from the bone marrow and its level in peripheral blood increases during mobilization due to activation of complement cascade/membrane attack complex. *Leukemia* 24 976–985. 10.1038/leu.2010.53 20357827 PMC2946378

[B77] Rauner M. Sipos W. Thiele S. Pietschmann P. (2013). Advances in osteoimmunology: pathophysiologic concepts and treatment opportunities. *Int. Arch. Allergy Immunol.* 160 114–125. 10.1159/000342426 23018236

[B78] Redlich K. Smolen J. S. (2012). Inflammatory bone loss: pathogenesis and therapeutic intervention. *Nat. Rev. Drug Discov.* 11 234–250. 22378270 10.1038/nrd3669

[B79] Roberts S. J. van Gastel N. Carmeliet G. Luyten F. P. (2015). Uncovering the periosteum for skeletal regeneration: the stem cell that lies beneath. *Bone* 70 10–18. 10.1016/j.bone.2014.08.007 25193160

[B80] Roelofsen T. Akkers R. Beumer W. Apotheker M. Steeghs I. van de Ven J. (2008). Sphingosine-1-phosphate acts as a developmental stage specific inhibitor of platelet-derived growth factor-induced chemotaxis of osteoblasts. *J. Cell. Biochem.* 105 1128–1138. 10.1002/jcb.21915 18819098

[B81] Rutherford C. Childs S. Ohotski J. McGlynn L. Riddick M. MacFarlane S. (2013). Regulation of cell survival by sphingosine-1-phosphate receptor s1p1 *via* reciprocal erk-dependent suppression of bim and pi-3-kinase/protein kinase c-mediated upregulation of mcl-1. *Cell Death Dis.* 4:e927. 10.1038/cddis.2013.455 24263101 PMC3847331

[B82] Ryland L. K. Fox T. E. Liu X. Loughran T. P. Kester M. (2011). Dysregulation of sphingolipid metabolism in cancer. *Cancer Biol. Ther.* 11 138–149. 10.4161/cbt.11.2.14624 21209555

[B83] Ryu J. Kim H. J. Chang E. J. Huang H. Banno Y. Kim H. H. (2006). Sphingosine 1-phosphate as a regulator of osteoclast differentiation and osteoclast-osteoblast coupling. *EMBO J.* 25 5840–5851. 10.1038/sj.emboj.7601430 17124500 PMC1698879

[B84] Sato C. Iwasaki T. Kitano S. Tsunemi S. Sano H. (2012). Sphingosine 1-phosphate receptor activation enhances bmp-2-induced osteoblast differentiation. *Biochem. Biophys. Res. Commun.* 423 200–205. 10.1016/j.bbrc.2012.05.130 22659743

[B85] Serra-Vinardell J. Roca-Ayats N. De-Ugarte L. Vilageliu L. Balcells S. Grinberg D. (2020). Bone development and remodeling in metabolic disorders. *J. Inherited Metab. Dis.* 43 133–144. 30942483 10.1002/jimd.12097

[B86] Soltanoff C. S. Yang S. Chen W. Li Y. P. (2009). Signaling networks that control the lineage commitment and differentiation of bone cells. *Crit. Rev. Eukaryot. Gene Expr.* 19 1–46. 10.1615/critreveukargeneexpr.v19.i1.10 19191755 PMC3392028

[B87] Spiegel S. Milstien S. (2002). Sphingosine 1-phosphate, a key cell signaling molecule. *J. Biol. Chem.* 277 25851–25854. 10.1074/jbc.r200007200 12011102

[B88] Spiegel S. Milstien S. (2003). Sphingosine-1-phosphate: an enigmatic signalling lipid. *Nat. Rev. Mol. Cell Biol.* 4 397–407. 12728273 10.1038/nrm1103

[B89] Spiegel S. Milstien S. (2011). The outs and the ins of sphingosine-1-phosphate in immunity. *Nat. Rev. Immunol.* 11 403–415. 10.1038/nri2974 21546914 PMC3368251

[B90] Stoffel W. (1999). Functional analysis of acid and neutral sphingomyelinases *in vitro* and *in vivo*. *Chem. Phys. Lipids* 102 107–121. 10.1016/s0009-3084(99)00079-1 11001565

[B91] Stoffel W. Jenke B. Block B. Zumbansen M. Koebke J. (2005). Neutral sphingomyelinase 2 (smpd3) in the control of postnatal growth and development. *Proc. Natl. Acad. Sci. U.S.A.* 102 4554–4559. 10.1073/pnas.0406380102 15764706 PMC555473

[B92] Tafesse F. G. Ternes P. Holthuis J. C. (2006). The multigenic sphingomyelin synthase family. *J. Biol. Chem.* 281 29421–29425. 10.1074/jbc.R600021200 16905542

[B93] Takabe K. Paugh S. W. Milstien S. Spiegel S. (2008). “Inside-out” Signaling of sphingosine-1-phosphate: therapeutic targets. *Pharmacol. Rev.* 60 181–195. 10.1124/pr.107.07113 18552276 PMC2695666

[B94] Takuwa Y. (2002). Subtype-specific differential regulation of rho family g proteins and cell migration by the edg family sphingosine-1-phosphate receptors. *Biochim. Biophys. Acta* 1582 112–120. 12069818 10.1016/s1388-1981(02)00145-2

[B95] Taniguchi M. Okazaki T. (2014). The role of sphingomyelin and sphingomyelin synthases in cell death, proliferation and migration-from cell and animal models to human disorders. *Biochim. Biophys. Acta* 1841 692–703. 10.1016/j.bbalip.2013.12.003 24355909

[B96] Teixeira V. Costa V. (2016). Unraveling the role of the target of rapamycin signaling in sphingolipid metabolism. *Progr. Lipid Res.* 61 109–133. 10.1016/j.plipres.2015.11.001 26703187

[B97] Ternes P. Franke S. Zahringer U. Sperling P. Heinz E. (2002). Identification and characterization of a sphingolipid delta 4-desaturase family. *J. Biol. Chem.* 277 25512–25518. 10.1074/jbc.M202947200 11937514

[B98] van Gastel N. Stegen S. Eelen G. Schoors S. Carlier A. Daniels V. W. (2020). Lipid availability determines fate of skeletal progenitor cells *via* sox9. *Nature* 579 111–117. 10.1038/s41586-020-2050-1 32103177 PMC7060079

[B99] Vogel P. Donoviel M. S. Read R. Hansen G. M. Hazlewood J. Anderson S. J. (2009). Incomplete inhibition of sphingosine 1-phosphate lyase modulates immune system function yet prevents early lethality and non-lymphoid lesions. *PLoS One* 4:e4112. 10.1371/journal.pone.0004112 19119317 PMC2606024

[B100] Wen D. Androjna C. Vasanji A. Belovich J. Midura R. J. (2010). Lipids and collagen matrix restrict the hydraulic permeability within the porous compartment of adult cortical bone. *Ann. Biomed. Eng.* 38 558–569. 10.1007/s10439-009-9858-z 19967451 PMC2841703

[B101] Weske S. Vaidya M. Reese A. von Wnuck Lipinski K. Keul P. Bayer J. K. (2018). Targeting sphingosine-1-phosphate lyase as an anabolic therapy for bone loss. *Nat. Med.* 24 667–678. 10.1038/s41591-018-0005-y 29662200

[B102] Yoshikawa Y. Yoshizawa T. Domae E. Hirai Y. Kamada A. Okazaki T. (2019). Knockdown of sphingomyelin synthase 2 inhibits osteoclastogenesis by decreasing rankl expression in mouse primary osteoblasts. *Biomed. Res.* 40 189–196. 10.2220/biomedres.40.189 31597904

[B103] Yu H. (2016). Sphingosine-1-phosphate receptor 2 regulates proinflammatory cytokine production and osteoclastogenesis. *PLoS One* 11:e0156303. 10.1371/journal.pone.0156303 27224249 PMC4880337

[B104] Zaidi M. (2007). Skeletal remodeling in health and disease. *Nat. Med.* 13 791–801. 10.1038/nm1593 17618270

[B105] Zemann B. Kinzel B. Muller M. Reuschel R. Mechtcheriakova D. Urtz N. (2006). Sphingosine kinase type 2 is essential for lymphopenia induced by the immunomodulatory drug fty720. *Blood* 107 1454–1458. 10.1182/blood-2005-07-2628 16223773

[B106] Zhang H. Desai N. N. Olivera A. Seki T. Brooker G. Spiegel S. (1991). Sphingosine-1-phosphate, a novel lipid, involved in cellular proliferation. *J. Cell Biol.* 114 155–167. 10.1083/jcb.114.1.155 2050740 PMC2289065

[B107] Zhao H. Li X. Zhang D. Chen H. Chao Y. Wu K. (2018). Integrative bone metabolomics-lipidomics strategy for pathological mechanism of postmenopausal osteoporosis mouse model. *Sci. Rep.* 8:16456. 10.1038/s41598-018-34574-6 30405156 PMC6220250

